# DEVELOPING AND IMPLEMENTING ALIVIADO DEMENTIA CARE IN HOSPICES: CHALLENGES ENCOUNTERED AND LESSONS LEARNED

**DOI:** 10.1093/geroni/igz038.1325

**Published:** 2019-11-08

**Authors:** Abraham A Brody, Shih-Yin Lin, Catherine E Schneider, Alycia A Bristol, Kimberly E Convery, Victor Sotelo

**Affiliations:** 1 NYU Rory Meyers College of Nursing, New York, United States; 2 NYU Rory Meyers College of Nursing, New York, New York, United States

## Abstract

Hospice was originally developed to care for individuals with metastatic, solid-tumor cancers. While advanced ADRD is now the primary illness in approximately 19% of the hospice population and presents as a co-morbid condition in many more, little evidence-based work has been performed to retool hospice to care for persons with ADRD and their caregivers. Aliviado Dementia Care-Hospice Edition is a systems level change program consisting of hospice workforce training, an implementation toolbox, and agency-wide workflow changes. Aliviado seeks to improve the quality of life for persons with ADRD and their caregivers receiving hospice, focused specifically on BPSD and pain assessment and management. In developing a coalition of hospice agencies and implementing this pragmatic intervention, we discuss our solutions to overcoming a number of barriers, including varying electronic health records, performing culture change with a disseminated workforce, scaling to 25 hospices, and working with some hospices who lack experience performing research.

